# Molecular Research on Mental Disorders

**DOI:** 10.3390/ijms24087104

**Published:** 2023-04-12

**Authors:** Magdalena Sowa-Kućma, Katarzyna Stachowicz

**Affiliations:** 1 Department of Human Physiology, Institute of Medical Sciences, Medical College of Rzeszow University, Kopisto Street 2a, 35-310 Rzeszow, Poland; 2 Department of Neurobiology, Maj Institute of Pharmacology, Polish Academy of Sciences, Smętna 12, 31-343 Kraków, Poland

Mental disorders and substance use disorders affect approximately 13% of the world’s population. There are various types of mental disorders with different presentations. In general, all of these are characterized by a combination of abnormal thoughts, perceptions, behaviors, emotions, and relationships with others. Mental disorders include, among others, depression, bipolar disorder, anxiety disorders, schizophrenia and other psychoses, dementia, and developmental disorders (including autism), which vary in severity. Drug addiction is an equally serious social issue. The causal mechanisms underlying mental disorders may contain keys to their effective prevention and treatment, but these remain poorly understood. The authors of this Special Issue offer new solutions and insights into the subject and present the most recent research in search of mechanisms of mental illness and treatment options.

This Special Issue of the *International Journal of Molecular Sciences*, entitled “Molecular Research on Mental Disorders”, comprises a total of eight contributions: four original articles and four reviews providing the latest research findings on mental disorders, including schizophrenia, anxiety, and addictive substance use.

Schizophrenia is one of the fifteen most disabling diseases worldwide, and its causes are poorly understood. It is increasingly being recognized as a multi-system disorder. The high frequency of coexistence of metabolic disorders and schizophrenia was a compelling reason to investigate this issue further. As Goh et al. [1] discussed in detail, ample evidence points to the potential importance of oxytocinergic dysfunction in the triggering mechanisms of schizophrenia. Despite the fact that this issue currently has many limitations, it appears to be future-proof. 

Limited knowledge of the pathophysiology of schizophrenia is also reflected in the lack of endogenous disease markers. As a result, Li et al. [2] sought new markers of schizophrenia. They attributed a special role to the linc01930/cCAS axis. A long intergenic non-protein coding RNA 1930 (linc01930) on chromosome 1p21.3 was identified as a schizophrenia-associated lncRNA. The authors discovered a correlation between linc01930 and interferon beta in the context of schizophrenia.

Animal models are commonly used to mirror human mental disorders, including schizophrenia, as described by Białoń and Wąsik [3]. Despite the availability of various models (pharmacological, genetic, or neurodevelopmental) that induce schizophrenia-like symptoms in animals, each of them has certain limitations, primarily due to interspecies differences between rodents (or zebrafish) and humans. None of the models discussed fully reflect human schizophrenic conditions, and individual models are developed to mimic different patterns of symptoms. Thus, the choice of the model should be optimal in terms of the research objectives. Regardless, the use of animal models is crucial to the development of new pharmacotherapies for schizophrenia.

Anxiety is frequently associated with psychiatric disorders or exists as a separate disease entity. In the search for novel chemical compounds active in schizophrenia, Żmudzka et al. [4] documented the antipsychotic- and anxiolytic-like properties of a novel arylpiperazine alkyl derivative of salicylamide (JJGW08) in two pharmacological models in mice. The compound was characterized as an antagonist of dopamine D2 receptors and serotonin 5-HT1A, 5-HT2A, and 5-HT7 receptors, acting on each receptor with decreasing potency according to the order in which they are listed. The tested compound had no adverse effects in the form of locomotor coordination or catalepsy. The authors proposed new arylpiperazine alkyl derivatives of salicylamide as potential compounds for treating schizophrenia with anxiety.

Anxiety behaviors may be regulated via calcium/calmodulin-dependent protein kinase I gamma. Piechota et al. [5] documented that a virus-induced knock-down of Camk1g expression in the amygdala of mice induced an increase in anxiety behavior. Further studies have shown that Camk1g is regulated by the glucocorticoid receptor and stress in the amygdala.

Depression, which affects nearly 5% of the adult population, is another major mental health issue (also strongly associated with anxiety). Like schizophrenia, the pathogenesis of depression is multifactorial. In recent years, interest in the role of the glutamatergic system in the development of this disease, as well as the targeting of glutamatergic receptors, has increased. As a result, understanding the changes that occur at excitatory synapses is becoming increasingly important. Changes in the levels of postsynaptic proteins appear to be particularly important, since they determine both the qualitative and quantitative composition of synapses and, ultimately, the functioning of entire neuronal networks. Samojedny et al. [6] discuss this issue in detail.

Depression can be either a primary or secondary disorder. It is often experienced by patients following a stroke (post-stroke depression, or PSD), which may be the result of biochemical changes in the brain or a psychological reaction to the losses caused by the stroke. Frank et al. [7] begin by discussing the relationship between PSD and post-stroke anxiety or post-stroke motor activity. Next, they review the potential mechanisms responsible for the development of PSD symptoms. Finally, they present a broad spectrum of possible pharmacological interventions, considering side effects. Among the most commonly used drugs for PSD, the authors list SSRIs, SNRIs, and TCAs, which are used as first-line treatments due to their effectiveness. Examples of additional drugs that have significantly boosted PSD patients’ outcomes include NMDA blockers and AMPA antagonists. The authors also consider non-pharmacological therapies and future therapies for PSD.

Cocaine use disorders are addressed separately in this Special Issue. The loss of control over substance use (including cocaine), compulsive drug seeking, and relapse are serious social issues. Gawlinski et al. [8] found that chronic intravenous cocaine administration impaired striatal and hippocampal expression of Wnt/β-catenin signaling in rats. The authors proposed Wnt signaling as a new target for potential cocaine use disorder drug therapy.

To summarize, this Special Issue provides the most recent and valuable information on the molecular basis of mental disorders, such as schizophrenia, anxiety, depression, and addiction.
